# Supplementary material for: A small molecule inhibitor of Rheb selectively targets mTORC1 signaling
Source: Nat Commun. 2018 Feb 7;9:548. doi: 10.1038/s41467-018-03035-z (PMC5803267; doi:10.1038/s41467-018-03035-z)
Supplement: Supplementary file 1 — Supplementary Information [file 41467_2018_3035_MOESM1_ESM.pdf]

**Supplementary Figures**

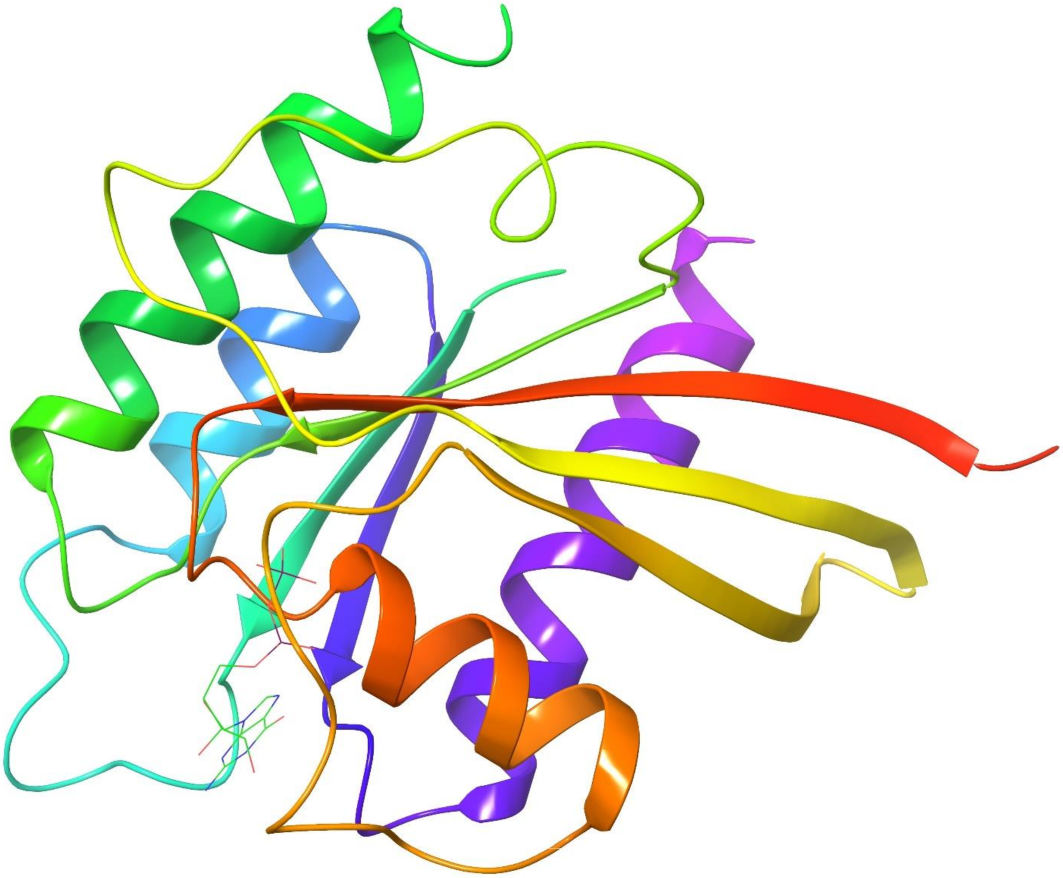

**Supplementary Figure 1. 2.05 Å X-ray crystal structure of GDP-bound Rheb solved in this study.** The switch II loop shown in yellow is in the closed state in the absence of any ligand.

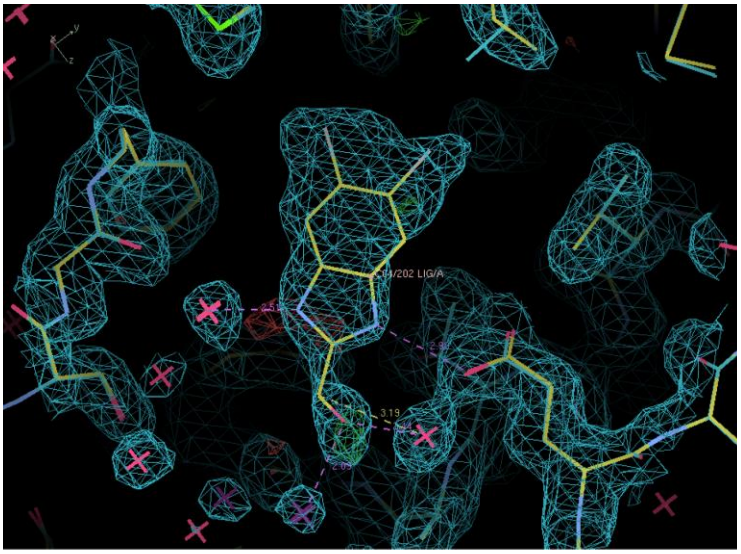

**Supplementary Figure 2. Experimental electron density observed in the switch II domain of Rheb crystals soaked with 1.**

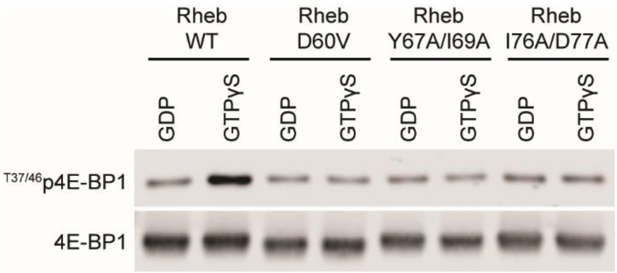


**Supplementary Figure 3. Comparison of WT and mutant forms of Rheb in Rheb-IVK.** The Rheb-IVK assay was performed with wild-type Rheb (WT) and Rheb mutated at residues in and around the Switch II region.

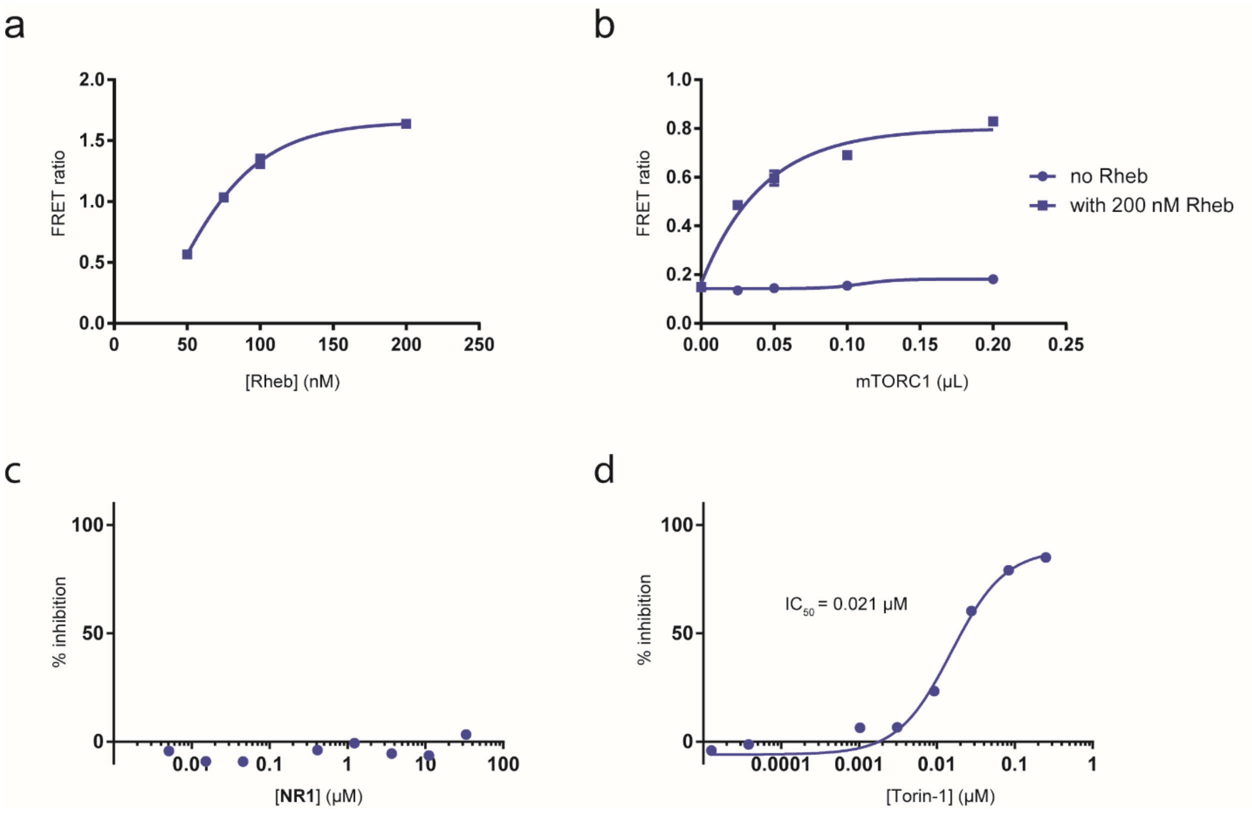


**Supplementary Figure 4. Optimization of Rheb-IVK and compound test in counter-screening assay.** (a) Dependence of FRET signal on Rheb concentration. (b) Dependence of FRET signal on amount of mTORC1 added. (c, d) **NR1** and Torin-1 were assessed for direct inhibition of mTOR kinase in a radiometric HotSpot assay. % inhibition is defined by maximum inhibition with control compound PI-103. Graphs represent data from one of at least two separate experiments.

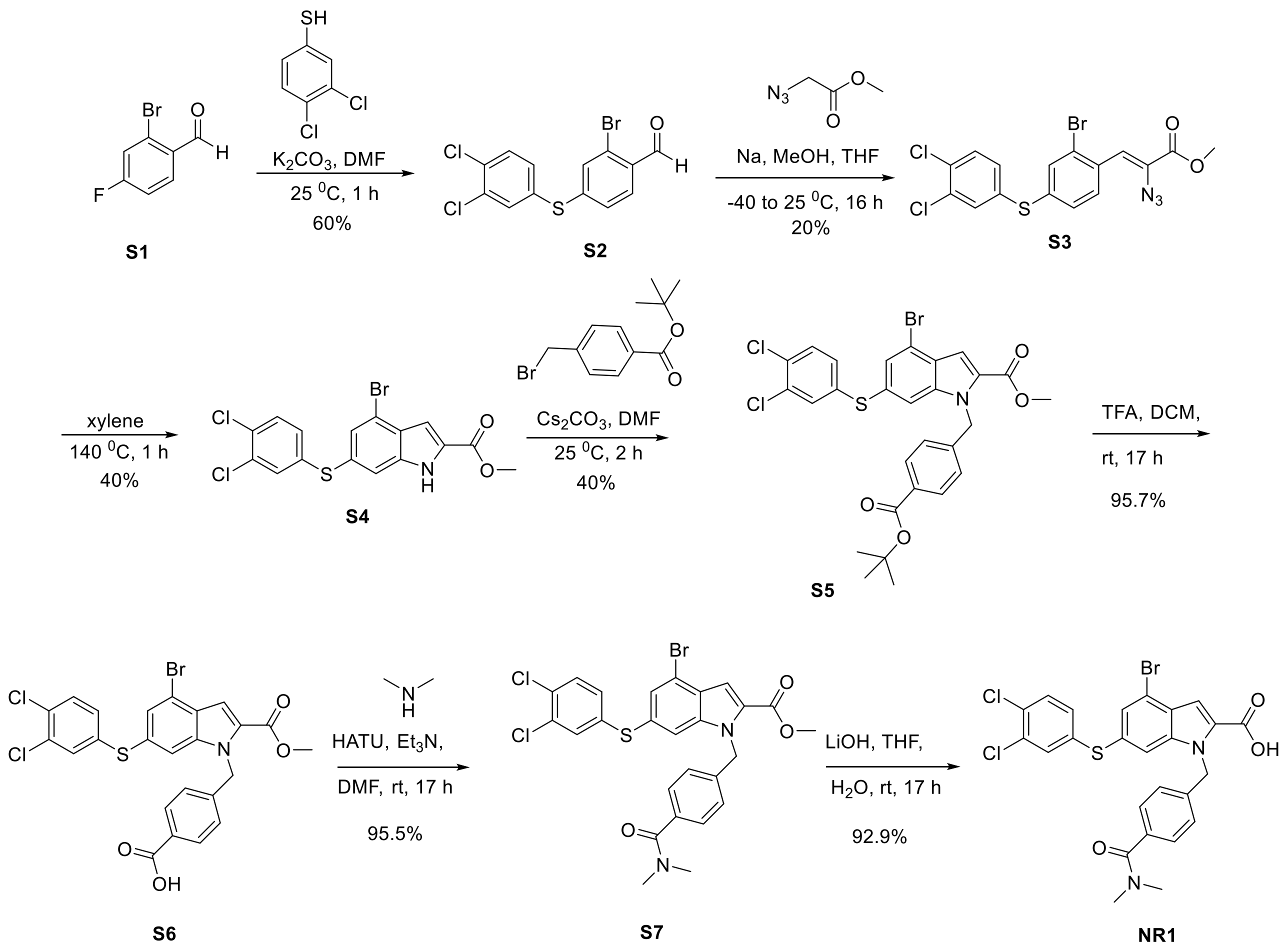


**Supplementary Figure 5. Synthesis of NR1**. Experimental procedures are detailed in the Methods and Supplementary Methods section of the manuscript.

a

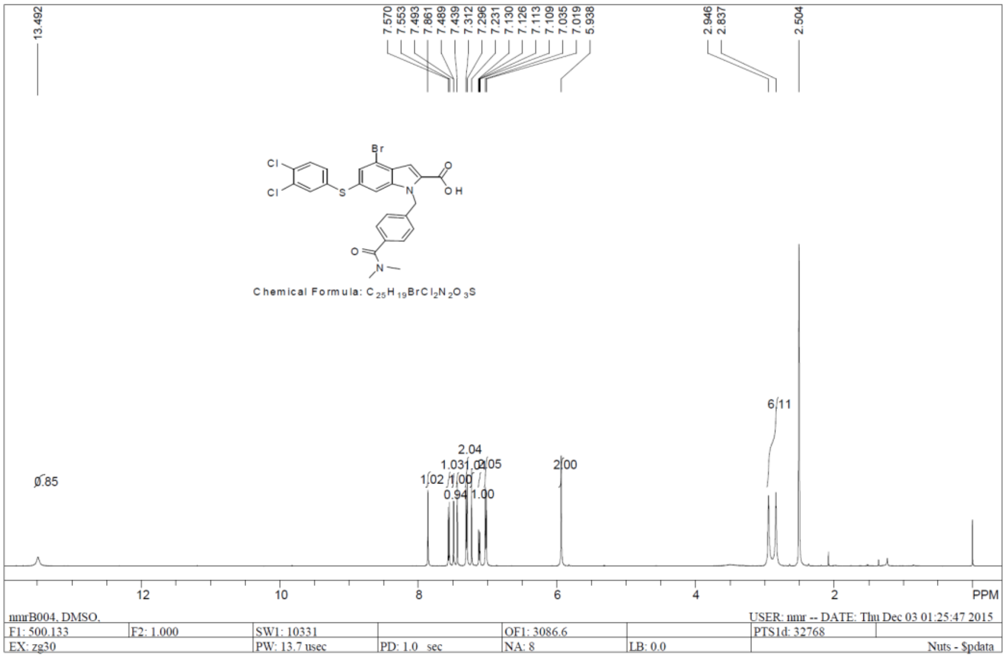


b

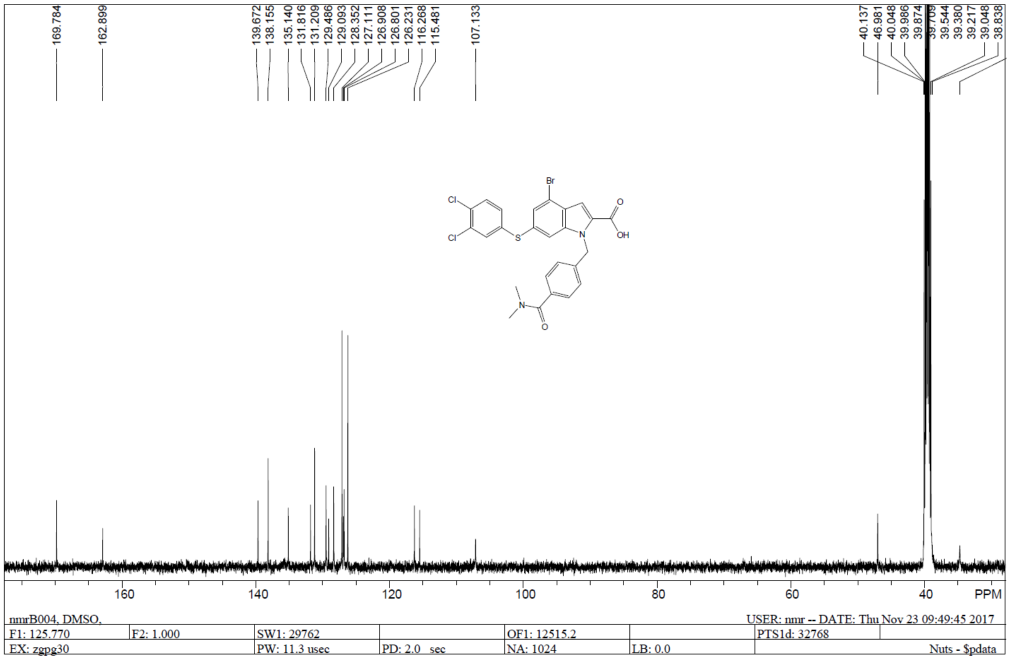


**Supplementary Figure 6. $^{1}$H and $^{13}$C NMR spectra of NR1**. (a) $^{1}$H-NMR and (b) $^{13}$C-NMR spectra for **NR1**.

a b

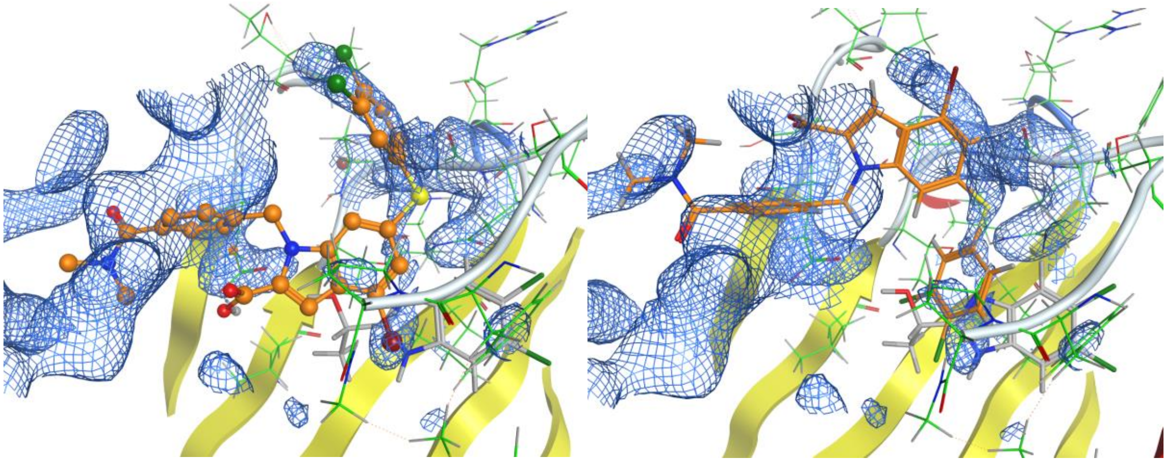

**Supplementary Figure 7. Experimental electron density observed in the switch II domain of Rheb crystals soaked with NR1.** Two distinct binding poses (a and b) were considered. Pose a fits the data better and is consistent with the structure-activity relationships observed for compounds in the series.

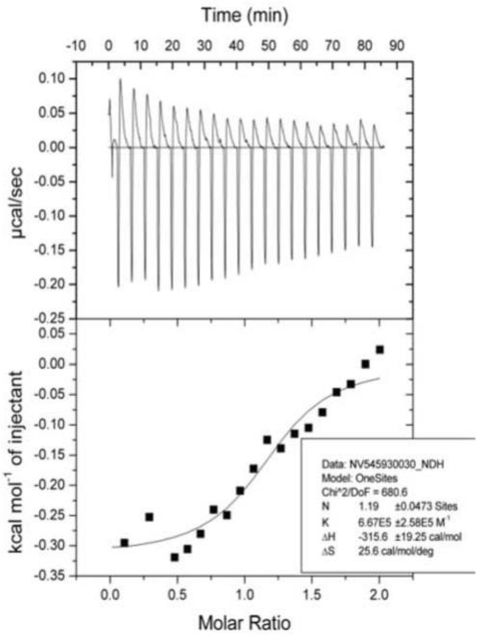


**Supplementary Figure 8. Measurement of binding constant of NR1 with Rheb using isothermal calorimetry.** $K_D$ = 1.5 µM.

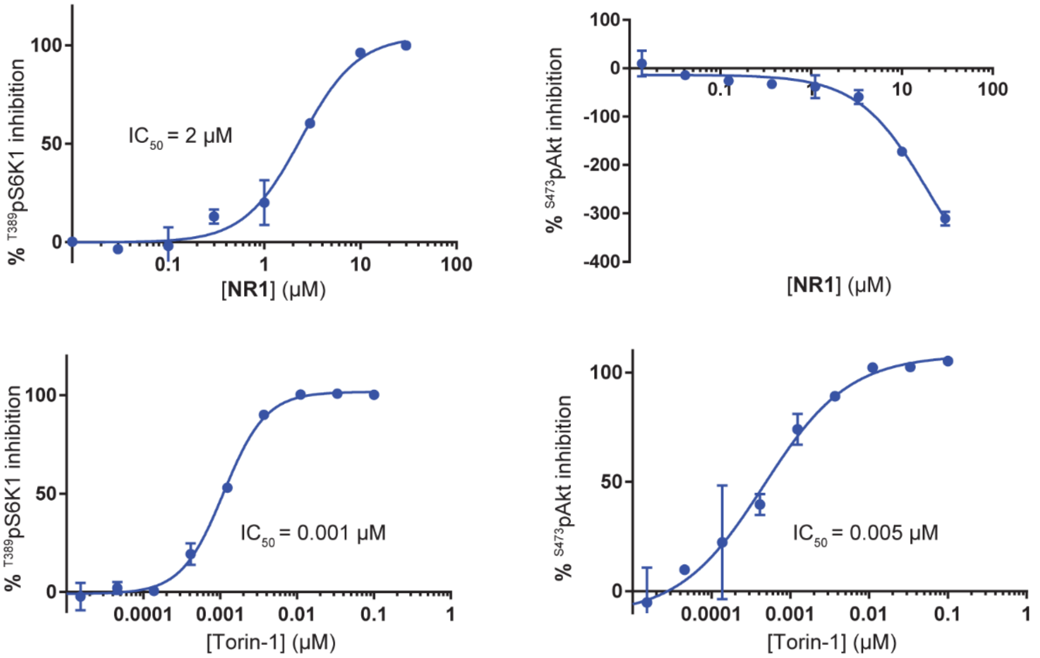


**Supplementary Figure 9. Performance of NR1 in $^{T389}$pS6K1 and $^{S473}$pAkt AlphaLISA assays.** Evaluation of **NR1** in MCF-7 under replete conditions. MCF-7 cells were treated with compounds for 120 min. The indicated phosphoproteins were detected using AlphaLISA assays for $^{T389}$pS6K1 (reflecting mTORC1 activity) and $^{S473}$pAKT (reflecting mTORC2 activity). Error bars reflect standard deviation of duplicates; graphs represent data for one of at least three separate experiments.

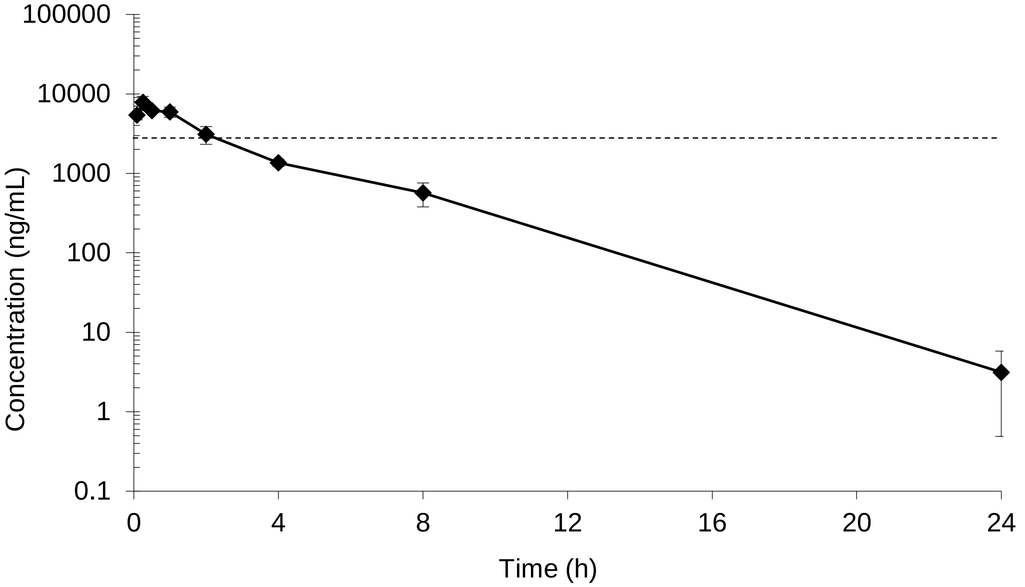


| PK parameter | Value |
|---|---|
| $T_{max}$ | 0.25 h |
| $C_{max}$ | 7803 ng $mL^{-1}$ |
| Regression time | 4~24 h |
| Terminal $t_{1/2}$ | 2.24 h |
| $AUC_{last}$ | 23486 h• ng $mL^{-1}$ |
| $AUC_{INF}$ | 23496 h• ng $mL^{-1}$ |

**Supplementary Figure 10. Pharmacokinetic profile of NR1 in mouse plasma after a single 30 mg/Kg (body weight) intraperitoneal dose**. The dotted line corresponds to 5 µM concentration. Error bars represent standard deviation of the mean values for n=3 mice.

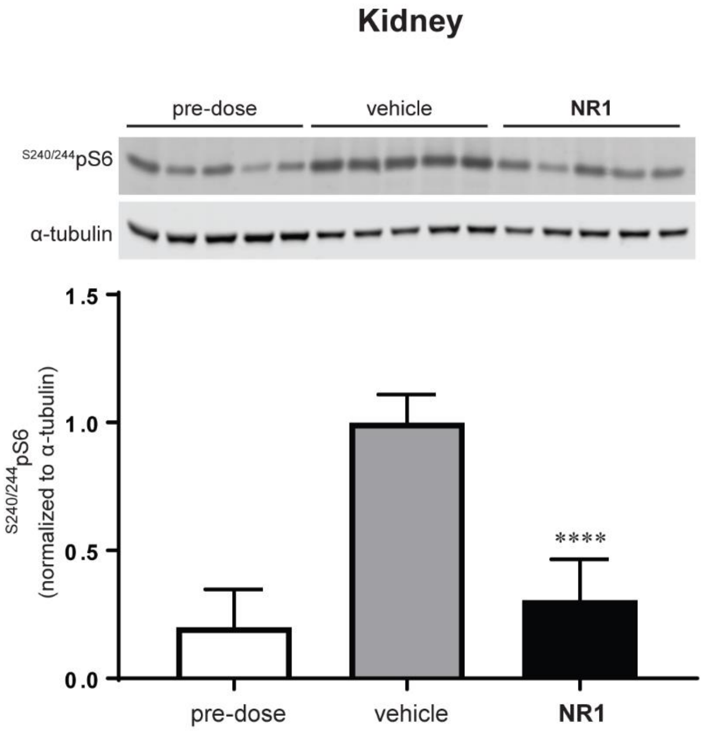


**Supplementary Figure 11. NR1 inhibits feeding-induced mTORC1 activation in kidney.** Mice were starved for 16 h pre-dose to achieve a basal level of mTORC1 activity, then dosed with **NR1** (30 mg $kg^{-1}$ IP) or vehicle and allowed to re-feed *ad libitum*. After 2 h, the kidney was collected, and $^{S240/244}$pS6 levels were measured by Western blot and quantified. The results are shown as mean ±SEM (n=5 per group). One way ANOVA for **NR1** with respect to vehicle are as follows: $F (2,12) = 47.69$, $p < 0.001$.

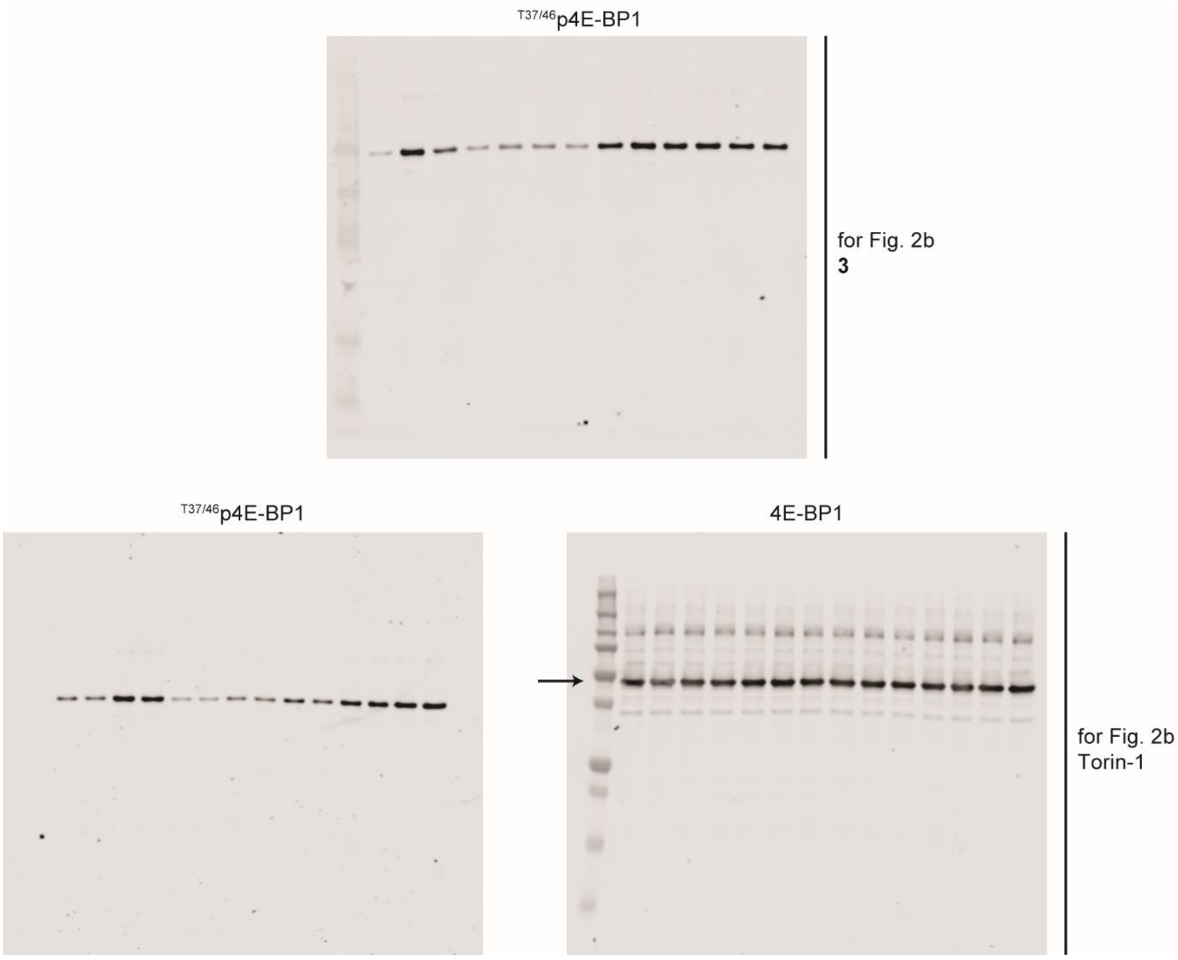


**Supplementary Figure 12. Full gel images for Figure 2.**

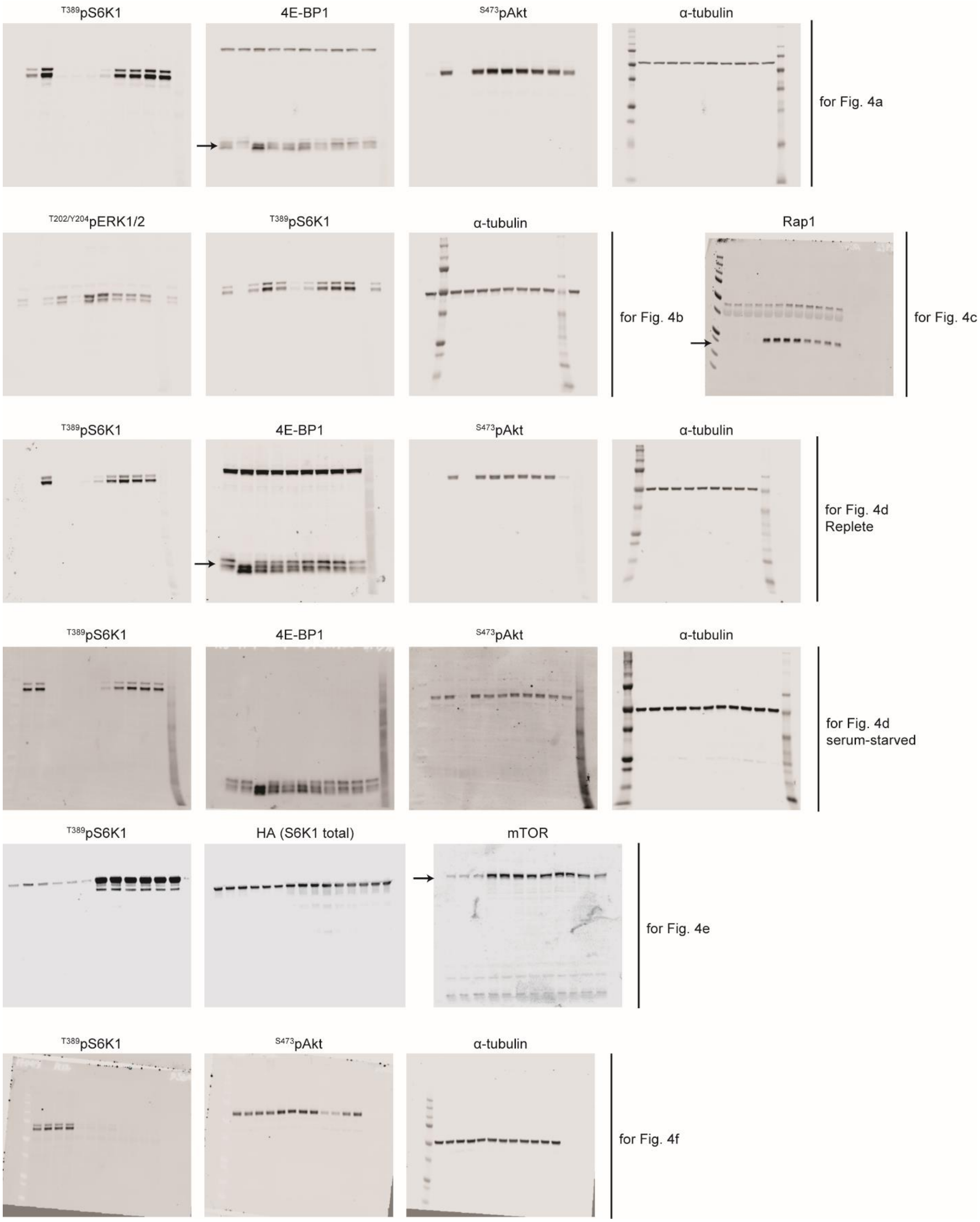


**Supplementary Figure 13. Full gels for Figure 4.**

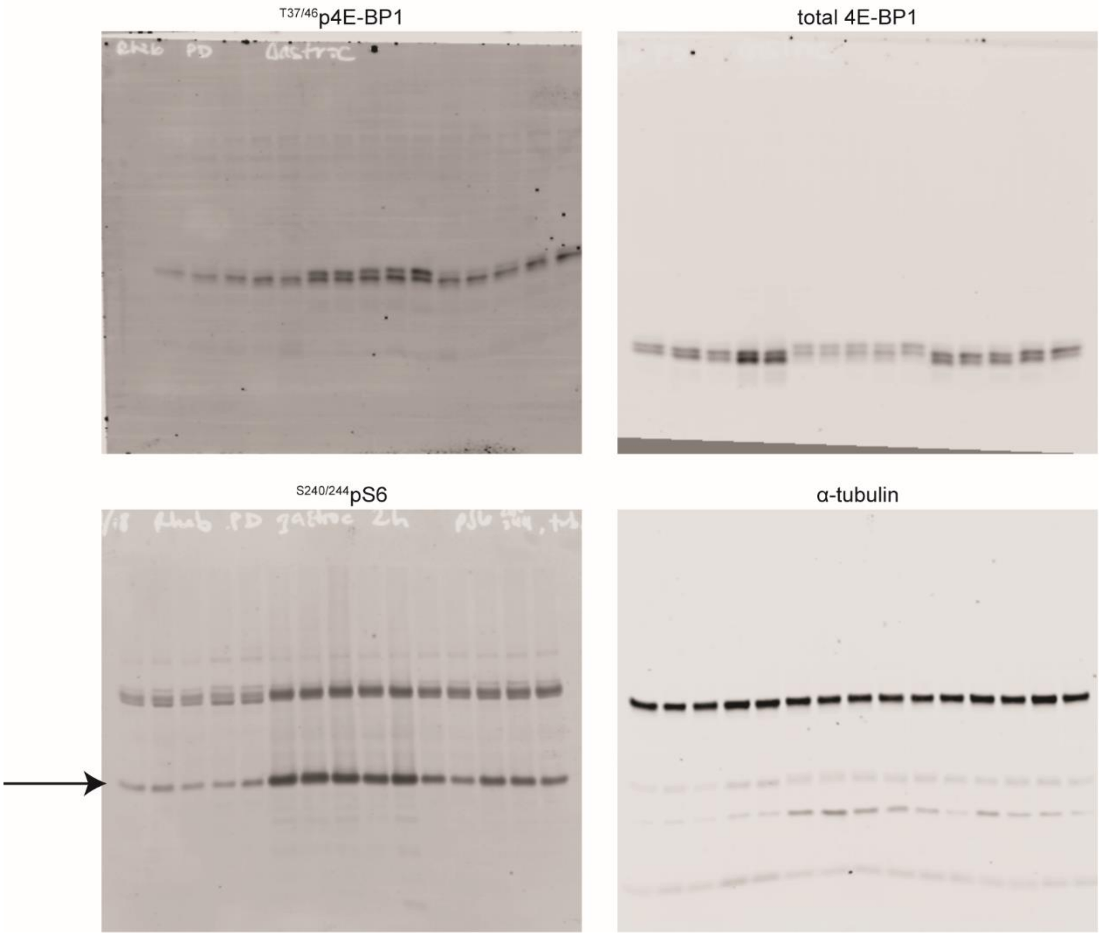


**Supplementary Figure 14. Full gels for Figure 6.** Full gels for skeletal muscle PD.

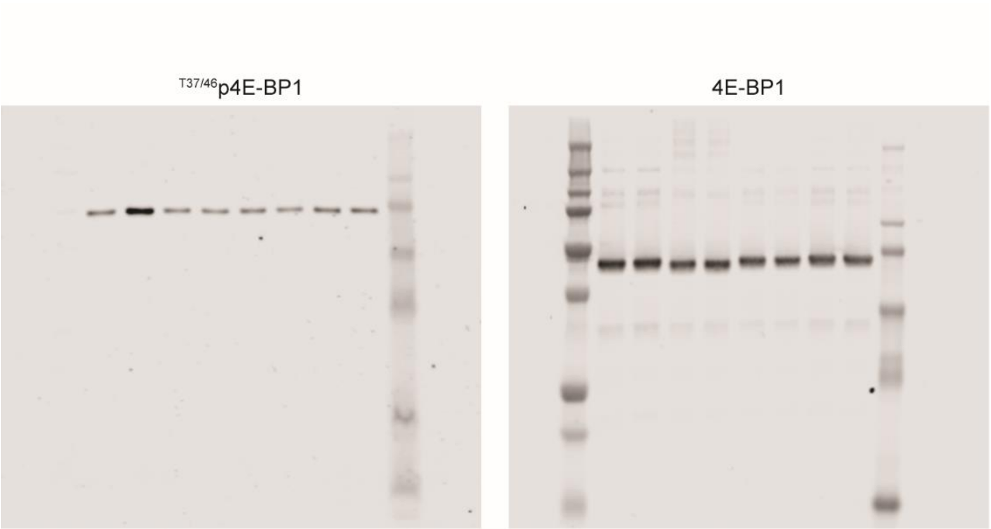


**Supplementary Figure 15. Full gels for Supplementary Figure 3.** Full gels for Rheb-IVK assay with Rheb mutants.

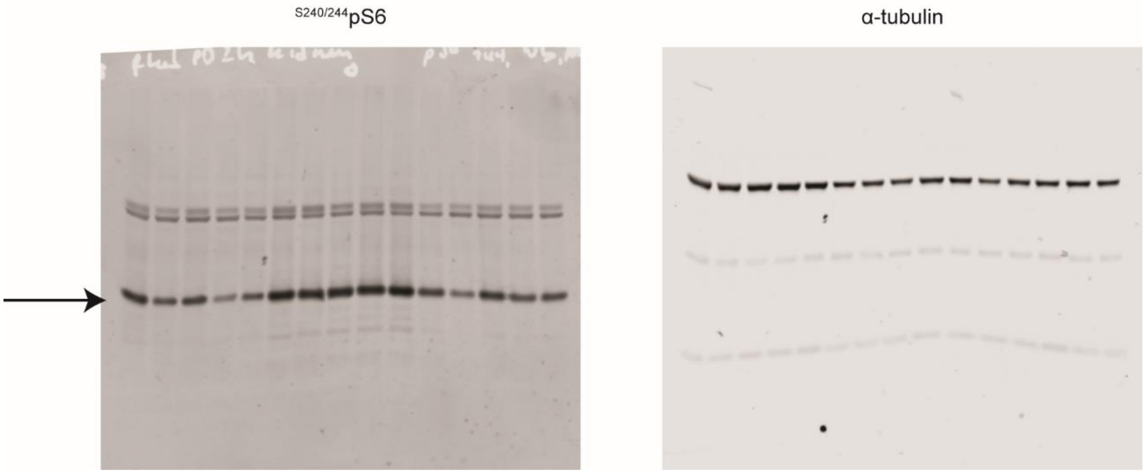


**Supplementary Figure 16. Full gels for Supplementary Figure 11.** Full gels for kidney PD.

## Supplementary Tables

| | Rheb + **NR1** |
|---|---|
| **Data collection** | |
| Space group | P1 |
| Cell dimensions | |
| $a$, $b$, $c$ (Å) | 49.15, 60.14, 66.32 |
| $\alpha$, $\beta$, $\gamma$ (°) | 68.35, 90.01, 72.05 |
| Resolution (Å) | 46.4-2.3 (2.36-2.3) |
| $R_{meas}$ | 0.204 (0.926) |
| $I$ / $\sigma I$ | 5.09 (1.25) |
| Completeness (%) | 93.0 (95.5) |
| Redundancy | 1.98 |
| | |
| **Refinement** | |
| Resolution (Å) | 46.4-2.6 |
| No. reflections | 17919 |
| $R_{work}$ / $R_{free}$ | 0.216 / 0.312 |
| No. atoms | |
| Protein | 5585 |
| Ligand/GDP/ion | 102/28/1 |
| Water | 18 |
| $B$-factors | |
| Protein | 36.5 |
| Ligand/GDP/ion | 144.3/22.7/24.0 |
| Water | 20.1 |
| R.m.s. deviations | |
| Bond lengths (Å) | 0.012 |
| Bond angles (°) | 1.646 |

*Values in parentheses are for highest-resolution shell.

**Supplementary Table 1. Data collection and refinement statistics (molecular replacement) for X-ray structure of NR1 bound to Rheb.**

**Supplementary Methods**

**Synthesis of NR1.** Synthesis of **NR1** is summarized in Supplementary Fig. 5. $^{1}$H and $^{13}$C NMR traces for **NR1** are depicted in Supplementary Fig. 6.

**S2.** 2-bromo-4-(3,4-dichlorophenylthio)benzaldehyde. A mixture of 2-bromo-4-fluorobenzaldehyde (7.45 g, 36.9 mmol), 3,4-dichlorobenzenethiol (6.89 mg, 38.7 mmol) and $K_2CO_3$ (6.1 g, 44.3 mmol) in DMF (120 mL) was stirred for 1 h at 25 °C. The reaction was quenched with water (250 mL) and extracted with ethyl acetate (300 mL). The organic phase was washed water (80 mL x 2), and brine (80 mL), dried ($Na_2SO_4$), filtered and concentrated *in vacuo* and the residue was purified by chromatography (silica, ethyl acetate/petroleum ether =1/20) to afford 2-bromo-4-(3,4-dichlorophenylthio)benzaldehyde (9 g, 25 mmol, 60%) as a white solid. $^{1}$H NMR (500 MHz, $CDCl_3$) δ 10.26 (s, 1H), 7.79 (d, *J* = 10 Hz, 1H), 7.61 (d, *J* = 2.5 Hz, 1H), 7.51 (d, *J* = 10 Hz, 1H), 7.38 (d, *J* = 2 Hz, 1H), 7.34 (dd, *J* = 10 Hz, *J* = 2.5 Hz, 1H), 7.15 (dd, *J* = 10 Hz, *J* = 1 Hz, 1H). S2 has been previously reported in the literature.[1]

**S3.** (*Z*)-methyl 2-azido-3-(2-bromo-4-(3,4-dichlorophenylthio)phenyl)acrylate. Na metal (184 mg, 8 mmol) was dissolved in dry MeOH (20 mL). A mixture of 2-bromo-4-(3,4-dichlorophenylthio)benzaldehyde (720 mg, 2 mmol) and methyl 2-azidoacetate (920 mg, 8 mmol) in dry THF (3 mL) was added at -40 °C. The reaction was stirred for 1 h at -40 °C and for 16 h at 25 °C under a $N_2$ atmosphere. The reaction was filtered. The cake was dried *in vacuo* to afford (*Z*)-methyl 2-azido-3-(2-bromo-4-(3,4-dichlorophenylthio)phenyl)acrylate (283 mg, 0.6 mmol, 20%) as an off-white solid. ESI-MS (EI+, m/z): 481.7 $[M+Na]^+$ and 430.8 $[M-N_2]^+$. $^{1}$H NMR (500 MHz, DMSO-*d6*) δ 8.16 (d, *J* = 8.5 Hz, 1H), 7.74-7.66 (m, 3H), 7.41-7.35 (m, 2H), 7.04 (s, 1H). $^{13}$C NMR (125 MHz, DMSO) δ 53.4, 120.7, 125.1, 128.0, 129.0, 131.2, 131.7, 131.8, 131.9, 132.2, 133.0, 137.6, 133.2, 133.9, 162.8.

**S4.** Methyl 4-bromo-6-(3,4-dichlorophenylthio)-1*H*-indole-2-carboxylate. A solution of (*Z*)-methyl 2-azido-3-(2-bromo-4-(3,4-dichlorophenylthio)phenyl)acrylate (250 mg, 0.55 mmol) in xylene (10 mL) was stirred for 1 h at 140 °C. The reaction was filtered. The cake was dried *in vacuo* to afford methyl 4-bromo-6-(3,4-dichlorophenylthio)-1*H*-indole-2-carboxylate (120 mg, 0.28 mmol, 40%) as a light yellow solid. ESI-MS (EI-, m/z): 427.8 and 429.8 $[M-H]^-$. $^{1}$H NMR (400 MHz, DMSO-$d_6$) δ 12.51 (s, 1H), 7.61 (d, *J* = 8.5 Hz, 1H), 7.56 – 7.53 (m, 2H), 7.39 (d, *J* = 0.9 Hz, 1H), 7.20 (dd, *J* = 8.5, 2.1 Hz, 1H), 7.06 (s, 1H), 3.90 (s,

3H). $^{13}$C NMR (125 MHz, DMSO) δ 52.2, 107.0, 115.9, 116.5, 126.4, 127.4, 129.1, 129.3, 129.6, 130.4, 131.4, 132.0, 137.3, 137.4, 161.0.

**S5.** Methyl 4-bromo-1-(4-(*tert*-butoxycarbonyl)benzyl)-6-(3,4-dichlorophenylthio)-1*H*-indole-2-carboxylate. A mixture of methyl 4-bromo-6-(3,4-dichlorophenylthio)-1*H*-indole-2-carboxylate (120 mg, 0.28 mmol), *tert*-butyl 4-(bromomethyl)benzoate (91 mg, 0.34 mmol) and $Cs_2CO_3$ (184 mg, 0.56 mmol) in DMF (5 mL) was stirred for 2 h at 25 °C. The reaction was quenched with water (10 mL) and extracted with ethyl acetate (30 mL). The organic phase was washed with water (10 mL x 2) and brine (10 mL), dried ($Na_2SO_4$), filtered and concentrated *in vacuo* and the residue was purified by chromatography (silica, ethyl acetate/petroleum ether =1/5) to afford methyl 4-bromo-1-(4-(*tert*-butoxycarbonyl)benzyl)-6-(3,4-dichlorophenylthio)-1*H*-indole-2-carboxylate (100 mg, 0.16 mmol, 40%) as a white solid. ESI-MS (EI+, m/z): 641.8 and 643.8 $[M+Na]^+$. $^1$H NMR (400 MHz, $CDCl_3$) δ 7.9-7.88 (d, *J* = 8.4 Hz, 2H), 7.4 (s, 1H), 7.35 – 7.26 (m, 4H), 7.04 – 6.99 (m, 3H), 5.83 (s, 2H), 3.9 (s, 3H), 1.56 (s, 9H). $^{13}$C NMR (100 MHz, $CDCl_3$) δ 28.2, 48.2, 52.1, 81.1, 111.4, 113.9, 117.6, 125.9, 127.0, 127.4, 128.7, 129.0, 130.0, 130.9, 131.2, 131.2, 131.4, 131.6, 133.3, 136.5, 139.4, 141.7, 161.7, 165.3.

**S6.** 4-((4-Bromo-6-(3,4-dichlorophenylthio)-2-(methoxycarbonyl)-1*H*-indol-1-yl)methyl)benzoic acid. A solution of methyl 4-bromo-1-(4-(*tert*-butoxycarbonyl)benzyl)-6-(3,4-dichlorophenylthio)-1*H*-indole-2-carboxylate (230 mg, 0.37 mmol) in DCM (8 mL) and TFA (4 mL) was stirred at RT for 17 h then filtered and concentrated *in vacuo* to afford 4-((4-bromo-6-(3,4-dichlorophenylthio)-2-(methoxycarbonyl)-1*H*-indol-1-yl)methyl)benzoic acid (200 mg, 95.7%) as a yellow solid which was used directly in the next step without further purification. ESI-MS ($EI^-$, m/z): 563.9 $[M-H]^-$. $^1$H NMR (400 MHz, DMSO-$d_6$) δ 12.91 (s, 1H), 7.86-7.84 (m, 3H), 7.56-7.52 (m, 1H), 7.48-7.45 (m, 2H), 7.28 (s, 1H), 7.21-7.07 (m, 3H), 5.9 (s, 2H), 3.85 (s, 3H). $^{13}$C NMR (100 MHz, DMSO) δ 47.6, 52.2, 109.9, 115.7, 116.2, 126.1, 126.2, 127.1, 128.9, 129.2, 129.6, 129.7, 130.2, 130.3, 131.3, 131.3, 131.9, 136.1, 137.0, 139.0, 142.7, 160.8, 166.9.

**S7.** Methyl 4-bromo-6-(3,4-dichlorophenylthio)-1-(4-(dimethylcarbamoyl)benzyl)-1*H*-indole-2-carboxylate. To a solution of 4-((4-bromo-6-(3,4-dichlorophenylthio)-2-(methoxycarbonyl)-1*H*-indol-1-yl)methyl)benzoic acid (130 mg, 0.23 mmol) in DMF (8ml) was added dimethylamine (40.7 mg, 0.69 mmol), $Et_3N$ (69.7 mg, 0.69 mmol) and HATU (262.2 mg, 0.69 mmol). The mixture was stirred at RT for 17 h then concentrated

and purified by prep-HPLC to afford methyl 4-bromo-6-(3,4-dichlorophenylthio)-1-(4-(dimethylcarbamoyl)benzyl)-1*H*-indole-2-carboxylate (130 mg, 95.5%) as a white solid. MS (EI+, m/z): 592.9 $[M+H]^+$ and 614.8 $[M+Na]^+$. $^1$H NMR (400 MHz, $CDCl_3$) δ 7.4 (s, 1H), 7.37-7.31 (m, 6H), 7.07-6.98 (m, 3H), 5.8 (s, 2H), 3.89 (s, 3H), 3.09 (s, 3H), 2.94 (s, 3H). $^{13}$C NMR (100 MHz, $CDCl_3$) δ 35.4, 39.6, 48.1, 52.1, 111.4, 114.0, 117.5, 126.1, 126.2, 127.0, 127.5, 127.6, 127.7, 128.7, 129.0, 131.0, 131.1, 131.4, 131.9, 133.2, 135.5, 136.7, 138.7, 139.4, 161.7, 171.2.

**Rheb-independent mTOR in vitro kinase assay (HotSpot).** For the Rheb-independent direct mTOR kinase assay, 3 μM N-terminal His-tagged human 4E-BP1 (Genbank Accession #NM_004095) was incubated with recombinant human mTOR (Genbank Accession #NP_004949.1, aa 1360-2549, N-terminal GST-tagged, expressed in insect cells) in reaction buffer (20 mM HEPES pH 7.5, 10 mM $MgCl_2$, 2 mM $MnCl_2$, 1 mM EGTA, 0.02% Brij35, 0.02 mg $ml^{-1}$ BSA, 0.1 mM $Na_3VO_4$, 2 mM DTT, 2% DMSO). The compounds were added to the kinase reaction mixture by Acoustic technology (Echo550; nanoliter range) and incubated for 20 min at room temperature before the addition of 100 μM $^{33}$P-ATP. The reaction was incubated for 2 h at room temperature, and kinase activity was detected by a P81 filter-binding method.

**AlphaLISA assays.** For AlphaLISA assays (Perkin Elmer #ALSU-PP70-A50K and #ALSU-PAKT-B50K), cells were plated on 96-well plates and treated for 120 min with compound before cell lysis. The AlphaLISA protocol was performed in 384-well plates as instructed by Perkin Elmer, and the plates were read on an EnVision plate reader. The maximum inhibition was defined by 1 μM Torin-1 treatment, which abolishes $^{T389}$pS6K1 and $^{S473}$pAkt in both AlphaLISA and Western formats.

**Supplementary References**

1. Ding D*, et al.* Discovery of novel benzoxaborole-based potent antitrypanosomal agents. *ACS Med Chem Lett* **1**, 165-169 (2010).
